# Potential Impact of 3% Hypertonic Saline Infusion on Tramadol Poisoning-Induced Electrocardiogram Changes; a Randomized Clinical Trial 

**DOI:** 10.22037/aaem.v10i1.1567

**Published:** 2022-04-13

**Authors:** Ali Omraninava, Ahmad Mehdizade, Ebrahim Karimi, Amir Ghabousian

**Affiliations:** 1 Emergency Department, Besat Hospital, AJA University of Medical Sciences, Tehran, Iran.; 2 Road Traffic Injury Research Center, Tabriz University of Medical Sciences, Tabriz, Iran.

**Keywords:** Poisoning, tramadol, saline solution, infusions, intravenous

## Abstract

**Introduction:**

Tramadol is a synthetic analgesic with weak mu-opioid receptor agonist activity. Tramadol overdose is associated with adverse cardiac effects due to inhibiting cardiac Na+ and K+ channels. This study aimed to investigate the potential ameliorative role of 3% hypertonic saline on the electrocardiogram (ECG) changes in patients presenting with tramadol poisoning.

**Methods:**

This was a single-center, controlled, randomized, single-blind clinical trial. Patients were randomized into the case (received hypertonic saline) and control (received placebo) groups. ECG was obtained twice in each group (upon arrival and following the intervention). Response to therapeutic interventions was evaluated using Wilcoxon Signed Ranks Test.

**Results:**

A total of 76 patients were included. The mean age of patients was 24.88 ± 4.29 years, and 62 (81.6%) were male. The mean ingested dose of tramadol was 1673.68 ± 608.85 (range: 550-2750) mg. The number needed to treat and the absolute risk reduction of 3% hypertonic saline in the treatment of wide QRS were 1 (95% CI: 1.00 – 1.00) and 100%, respectively. In the treatment of long QTc, these measures were 1.9 (95%CI: 1.2 – 4.5) and 53.85% (95%CI: 22.00 – 85.69), respectively.

**Conclusion:**

Given that hypertonic saline infusion can significantly ameliorate tramadol-mediated ECG changes, including QRS prolongation and QT lengthening, it can be regarded as a potential therapeutic strategy to prevent the development of life-threatening ventricular arrhythmias caused by tramadol toxicity.

## 1. Introduction:

Tramadol is a synthetic analgesic with weak mu-opioid receptor agonist activity and is commonly used to mitigate moderate to severe pain (1). It is well-established that tramadol hinders the reuptake of several neurotransmitters, including serotonin and norepinephrine (2). Even though tramadol has illustrated a low risk for dependence and abuse compared to other opioids, the incidence of tramadol-mediated toxicity and its complications have been on the rise over the past decades (3). Tramadol poisoning can adversely affect multiple organs throughout the body. One of the most fearsome ramifications of tramadol toxicity is its catastrophic impact upon cardiac action potential (4). According to the available studies, at high doses, tramadol can interfere with cardio-myocyte depolarization and repolarization via inhibiting voltage-gated Na^+^ and delayed rectifier k^+^ channels, respectively (5). Electrocardiogram (ECG) findings in patients with tramadol poisoning are mainly consistent with the Na^+^ channel blockade activity (6). 

The mainstay of tramadol toxicity treatment is general supportive care and possible seizure management (7). However, there is a lack of evidence with regard to reversing potentially life-threatening ECG changes in this regard. The use of hypertonic saline (HS) with the aim of sodium loading has been identified as a potential alternative method to sodium bicarbonate use in patients with tricyclic antidepressants (TCA) intoxication, which also associates with Na^+^ channel blockade (8). However, the impact of hypertonic saline on tramadol-induced ECG abnormalities has never been studied. This study aimed to investigate the potential impact of 3% HS on the ECG changes and associated complications in patients presenting with tramadol poisoning.

## 2. Methods:

***2.1. Study design and setting***

This is a controlled, randomized, single-blind clinical trial intending to evaluate the potential therapeutic role of 3% HS in ameliorating ECG changes and associated complications followed by tramadol toxicity. The study was conducted on patients presenting to Besat General Hospital, Tehran, Iran, from March through October 2021. The local ethics committee (Biomedical Research Ethics Committee) approved the protocol of present study (IR.AJAUMS.REC.1400.126) and it was registered in Iranian Registry of Clinical Trials (IRCT: 20220213054017N1) Written informed consent was obtained from patients or their next of kin and researchers acted solely based on the Helsinki Declaration.

***2.2. Participants***

**Figure 1:**
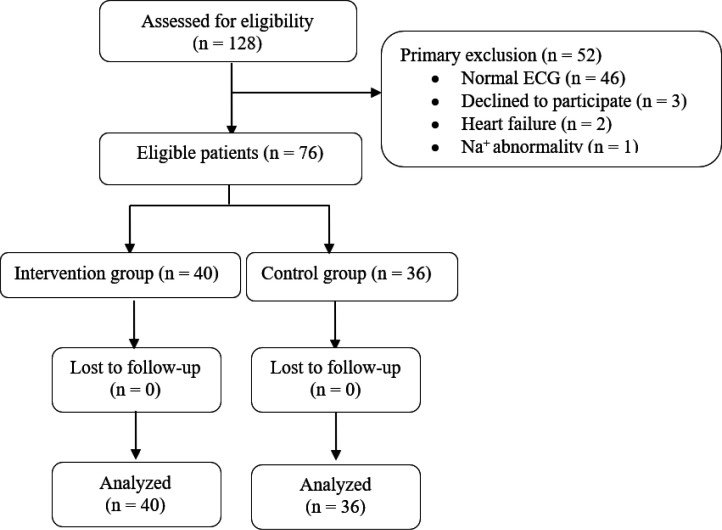
Flow diagram of patient allocation. ECG: electrocardiogram

Tramadol poisoned cases with at least one of the following ECG abnormalities were included: right bundle brunch block (RBBB), long QTc, Tall R wave, prolonged QRS interval, and right axis deviation. These parameters were selected based on the available data regarding the ECG manifestations of Na^+ ^and K^+ ^channel blockers (9, 10). Patients not willing to participate, those under 18 years of age, pregnant women, and patients with underlying cardiovascular disorders, including heart failure and cardiomyopathy, patients on antiarrhythmic or arrhythmogenic drugs, and those with liver failure, electrolyte disturbance (hypo- and hypernatremia), and chronic kidney disease, were excluded from the present study (Figure 1).

***2.3. Data gathering and therapeutic intervention***

The demographic characteristics (age and sex), medical history, physical findings upon arrival, drug dosage, and relevant complications (seizure, falling, shoulder dislocation, head trauma, and loss of consciousness) were gathered using a predesigned checklist. Eligible patients were randomly allocated to either case (intervention) or control (placebo) group using a simple randomization method. All patients were treated according to the current standard of care for acute tramadol poisoning. Patients in the control group, received 3 ml/kg (maximum dose:150 ml) of 3% dextrose water over 20 minutes as a placebo. However, patients in the intervention group received the intravascular infusion of 3% hypertonic saline (3 ml/kg over 20 minutes; maximum dose: 150 ml) in addition to the standard of care. ECG was obtained twice in each group (upon arrival and after completing the intervention). A 12-lead ECG was acquired by a registered nurse under the supervision of a senior emergency medicine resident using a Carewell Digital 12-channel ECG-1112M electrocardiograph. The interpretation of ECG was carried out by a cardiologist blinded to the study protocol. To this end, heart rate, QRS axis (normal between -30 to +100), QRS duration (normal< 110 ms), QTc interval (normal<0.44 ms), bundle branch block, and the presence of dominant R wave in aVR (R wave height greater than 3 mm or R/S ratio more than 0.7) were documented. Right bundle branch block (RBBB), long QTc, Tall R wave, wide QRS, and axis deviation were the studied ECG abnormalities. 

***2.4. Outcomes***

The primary outcome of the current study was the effects of 3% HS on ECG changes and the secondary outcome was its effect on other complications of tramadol poisoning.

***2.5. Statistical analysis***

The study analyses were performed using IBM® SPSS® Statistics 26. Normal distribution was assessed using Kolmogorov-Smirnov method. Data were presented as mean ± standard deviation or frequency (%). Taking into account normality, we used Independent-Samples T-Test and Mann-Whitney U Test to compare quantitative characteristics between the two groups, while we employed Chi-Square test for qualitative ones. Response to therapeutic intervention was evaluated using Wilcoxon Signed Ranks Test. In any case, we considered p-value less than 0.05 as significant.

## 3. Results:

***3.1. Baseline characteristics of studied cases***

A total of 76 patients with tramadol intoxication and ECG abnormality were enrolled during this clinical trial and were included in final analysis. The mean age of patients was 24.88 ± 4.29 years, and 62 (81.6%) were male. The mean ingested dose of tramadol was 1673.68 ± 608.85 (range: 550-2750) mg. Aspiration pneumonia was detected in 2 (2.6%) patients, signs of head trauma in 1 (1.3%), altered mental status upon arrival in 31 (32.3%), seizure in 37 (38.5%), and shoulder dislocation in 25 (32.9%). There was no statistically significant relationship between tramadol dosage and seizure occurrence (1657.14 ± 654.29 mg in patients who developed seizure and 1683.33 ± 587.66 mg in those who did not, p= 0.858). Out of 76 patients, 1 (1.3%) patient died despite resuscitation efforts. This patient had been randomly allocated to the control group. The intervention and control groups were similar regarding the mean age (24.53 ± 4.03 vs. 25.28 ± 4.58, respectively; p = 0.449), male/female ratio (33/7 vs. 29/7; respectively; p = 0.827), and ingested tramadol dose (1548.75 ± 635.43 vs. 1812.50 ± 553.86; respectively; p = 0.059). 

***3.2. ECG features***

Table 1 summarizes abnormal ECG findings upon arrival and after intervention in both study groups. The two groups had similar frequency of RBBB (p = 0.217), long QTc (p = 1.00), Tall R wave (p = 1.00), wide QRS (p = 606), and axis deviation (p = 0.864) in baseline ECG, while the frequency of wide QRS interval (p = 0.007) and long QTc (p = 0.025) was significantly lower in patients who received 3% hypertonic saline in the second ECG (table 1). The number needed to treat and the absolute risk reduction of 3% hypertonic saline in the treatment of wide QRS were 1 (95% CI: 1.00 – 1.00) and 100%, respectively. In the treatment of long QTc, these measures were 1.9 (95%CI: 1.2 – 4.5) and 53.85% (95%CI: 22.00 – 85.69), respectively.

**Table 1:** Comparison of baseline and post-intervention electrocardiogram (ECG) findings between case (3% hypertonic saline) and control (placebo) groups

ECG findings	Case (n=40)	Control (n=36)	P-value
Baseline			
**RBBB**	3 (7.5)	6 (16.7)	0.217
**Wide QRS**	5 (12.5)	6 (16.7)	0.606
**Long QTc**	13 (32.5)	13 (36.1)	1.00
**Tall R wave**	14 (35.0)	14 (38.9)	1.00
**Axis deviation**	17 (42.5)	16 (44.4)	0.864
Post-intervention			
**RBBB**	1 (2.5)	3 (8.3)	0.255
**Wide QRS**	0 (0)	6 (16.7)	0.007
**Long QTc**	4 (10.0)	11 (30.6)	0.025
**Tall R wave**	9 (22.5)	10 (27.8)	0.596
**Axis deviation**	11 (27.5)	9 (25.0)	0.805

Data are presented as number (%).; RBBB: right bundle branch block.

## 4. Discussion:

Based on the results of this clinical trial, 3% hypertonic saline administration in addition to the routine standard of care in patients with tramadol poisoning can ameliorate potentially life-threatening ECG changes, including QRS prolongation and QTc lengthening. Routine standard of care itself cannot result in statistically significant changes in the aforementioned parameters. It is noteworthy that after receiving hypertonic saline, the absolute risk reduction for QRS prolongation and QTc interval was 100% and 53.85%, respectively. This gap between the absolute risk reduction for QRS and QT interval, in part, is due to the fact that tramadol-induced cardiotoxicity is a combination of Na^+^ and K^+^ channels blockade. 

The QRS duration stands for the ventricular depolarization time and QTc interval mainly represents ventricular repolarization. However, given that QT interval comprises QRS complex, ST segment, and T wave, a widened QRS duration can give rise to QT prolongation without inducing any repolarization delay (11). An acquired prolonged QTc interval, which mostly occurs owing to the inhibition of the inward potassium rectifier (IKr) channels, is associated with a high risk of developing life-threatening arrhythmias, such as torsades de points (TdP)(12). Currently, magnesium sulfate is used for treating and preventing the recurrence of TdP. On the other hand, a widened QRS duration due to inhibition of Na^+^ channels, can also increase the possibility of developing ventricular arrhythmia (10, 13). 

Although the pros and cons of hypertonic saline administration in the setting of tramadol poisoning have not already been explored, its ameliorative role has been proven to be equal or superior to sodium bicarbonate in animals with TCA toxicity, which also leads to Na+ channel blockade (14, 15). Moreover, in our study, the administration of hypertonic saline did not contribute to any significant adverse effect. 

At present, the Drug Enforcement Administration has placed tramadol into Schedule IV of controlled substances, implying that it contains a low risk for dependence and abuse. However, opioid poisoning and abuse are not thin on the ground, particularly when it comes to the Middle East (16, 17). Published studies have demonstrated conflicting results regarding dose-dependency of seizure in patients with tramadol poisoning. A recent meta-analysis, in which 51 studies were included, has found that among patients presenting with tramadol overdose, not taking tramadol abusers into account, the drug dose was not significantly different among patients with or without seizure development (pooled Standardized Mean Difference: 0.27, CI 95%:0.15 to 0.7) (18). These findings were consistent with the current study results, which included patients with tramadol overdose. In harmony with our results, in a prospective study conducted on 1402 patients with tramadol poisoning, the most frequent abnormal ECG findings were sinus tachycardia (33%), QRS right axis deviation (24.2%), QRS widening (6.5%), long QTc interval (18.4%), and RBBB (5.2%) (19). 

Nonetheless, the management of cardiotoxicity induced by tramadol is complex, given the involvement of both Na^+^ and K^+^ channels. Furthermore, there is no consensus regarding the best approach to manage this condition. Our study shows that hypertonic saline infusion is an effective therapeutic strategy to mitigate tramadol-mediated ECG abnormalities. Lastly, our study had several limitations. It was carried out in a single center setting and included a limited number of patients. Further multicenter studies are necessary to prove the generalizability of these findings. 

## 5. Conclusion:

Given that hypertonic saline infusion can significantly ameliorate tramadol-mediated ECG changes, including QRS prolongation and QT lengthening, it can be regarded as a potential therapeutic strategy to prevent the development of ventricular arrhythmias caused by tramadol toxicity.

## 6. Declarations:

### 6.1. Acknowledgment

None.

### 6.2. Authors’ contributions

All the authors meet the standard authorship criteria based on the recommendations of international committee of medical journal editors.

### 6.3. Conflict of interest

None.

### 6.4. Funding and support

None.
